# Structural and Functional Analysis of Laninamivir and its Octanoate Prodrug Reveals Group Specific Mechanisms for Influenza NA Inhibition

**DOI:** 10.1371/journal.ppat.1002249

**Published:** 2011-10-20

**Authors:** Christopher J. Vavricka, Qing Li, Yan Wu, Jianxun Qi, Mingyang Wang, Yue Liu, Feng Gao, Jun Liu, Enguang Feng, Jianhua He, Jinfang Wang, Hong Liu, Hualiang Jiang, George F. Gao

**Affiliations:** 1 CAS Key Laboratory of Pathogenic Microbiology and Immunology, Institute of Microbiology, Chinese Academy of Sciences, Beijing, China; 2 School of Life Sciences, University of Science and Technology of China, Hefei, China; 3 Graduate University, Chinese Academy of Sciences, Beijing, China; 4 National Laboratory of Biomacromolecules, Institute of Biophysics, Chinese Academy of Sciences, Beijing, China; 5 State Key Laboratory of Drug Research, Shanghai Institute of Materia Medica, Shanghai Institutes for Biological Sciences, Chinese Academy of Sciences, Shanghai, China; 6 Shanghai Institute of Applied Physics, Chinese Academy of Sciences, Shanghai, China; 7 Beijing Institutes of Life Science, Chinese Academy of Sciences, Beijing, China; Johns Hopkins University - Bloomberg School of Public Health, United States of America

## Abstract

The 2009 H1N1 influenza pandemic (pH1N1) led to record sales of neuraminidase (NA) inhibitors, which has contributed significantly to the recent increase in oseltamivir-resistant viruses. Therefore, development and careful evaluation of novel NA inhibitors is of great interest. Recently, a highly potent NA inhibitor, laninamivir, has been approved for use in Japan. Laninamivir is effective using a single inhaled dose via its octanoate prodrug (CS-8958) and has been demonstrated to be effective against oseltamivir-resistant NA *in vitro*. However, effectiveness of laninamivir octanoate prodrug against oseltamivir-resistant influenza infection in adults has not been demonstrated. NA is classified into 2 groups based upon phylogenetic analysis and it is becoming clear that each group has some distinct structural features. Recently, we found that pH1N1 N1 NA (p09N1) is an atypical group 1 NA with some group 2-like features in its active site (lack of a 150-cavity). Furthermore, it has been reported that certain oseltamivir-resistant substitutions in the NA active site are group 1 specific. In order to comprehensively evaluate the effectiveness of laninamivir, we utilized recombinant N5 (typical group 1), p09N1 (atypical group 1) and N2 from the 1957 pandemic H2N2 (p57N2) (typical group 2) to carry out *in vitro* inhibition assays. We found that laninamivir and its octanoate prodrug display group specific preferences to different influenza NAs and provide the structural basis of their specific action based upon their novel complex crystal structures. Our results indicate that laninamivir and zanamivir are more effective against group 1 NA with a 150-cavity than group 2 NA with no 150-cavity. Furthermore, we have found that the laninamivir octanoate prodrug has a unique binding mode in p09N1 that is different from that of group 2 p57N2, but with some similarities to NA-oseltamivir binding, which provides additional insight into group specific differences of oseltamivir binding and resistance.

## Author Summary

The influenza neuraminidase (NA) enzyme is the most successful drug target against the seasonal and pandemic flu. The 2009 H1N1 flu pandemic led to record sales of the NA inhibitors oseltamivir (Tamiflu) and zanamivir (Relenza). Recently, a new drug, laninamivir (Inavir), has been approved for use in Japan can also be administered effectively using a single dose via its octanoate prodrug (CS-8958), however its effectiveness against oseltamivir-resistant influenza infection has not been demonstrated in clinical studies. In this study we comprehensively evaluate the effectiveness of laninamivir and its prodrug using NA from different groups with different active site features. We expressed and purified a group 2 NA from the 1957 pandemic H2N2, an atypical group 1 NA from the 2009 H1N1 pandemic and a group 1 NA from avian H12N5. NA inhibition was assayed and NAs were further crystallized with each inhibitor to determine the structural basis of their action. We found that laninamivir inhibition is highly potent for each NA, however binding and inhibition of laninamivir and its prodrug showed group specific preferences. Our results provide the structural and functional basis of NA inhibition using classical and novel inhibitors, with NAs from multiple serotypes with different properties.

## Introduction

The 2009 pandemic swine origin influenza A H1N1 virus (pH1N1) has reminded the world of the threat of pandemic influenza [1], [2], [3]. In 2009, the total sales of Tamiflu (oseltamivir phosphate) increased to over 3 billion US dollars (Annual General Meeting of Roche Holding Ltd, 2 March 2010). The total sales of Relenza (zanamivir) in 2009 were over 1 billion (GlaxoSmithKline Quarter 4 Report, 4 February 2010). Additionally, 5.65 million packs of Tamiflu were donated to the WHO in 2009 to replenish their stockpiles (Roche, Annual General Meeting of Roche Holding Ltd, 2 March 2010). Since the WHO has downgraded the threat of pH1N1 from the pandemic level in August 2010, there have still been ongoing reports of pH1N1 outbreaks in south-eastern states of the USA, India and New Zealand (US CDC). Furthermore, a new variant of pH1N1 has even been detected in Singapore, New Zealand and Australia [4]. Throughout the world, vaccinations have still been strongly advocated and stockpiles of oseltamivir and zanamivir are on reserve in case of another severe influenza outbreak in the near future. Both oseltamivir and zanamivir are excellent examples of modern structure-based drug-design and function as competitive inhibitors of the influenza neuraminidase (NA), and are by far the most commonly used influenza drugs [5], [6], [7], [8].

Influenza A virus contains two proteins on its surface in addition to the ion channel M2: hemagglutinin (HA) and NA [9]. Both M2 and NA are targets for clinically-available influenza drugs, however M2 drugs are rarely used anymore because M2 develops drug-resistant mutations very easily [10]. In the influenza virus infection life cycle, HA binds to terminally linked sialic acid receptors on the surface of host cells, allowing the virus to gain entry. In order for the influenza virus to efficiently break free from already infected cells and to continue replicating, sialic acid containing HA receptors must be destroyed. NA, which is a sialidase, catalyzes hydrolysis of terminally linked sialic acid and functions as the receptor-destroying element of influenza A and B viruses.

Influenza A NA has been grouped into 9 different serotypes, N1-N9, based upon antigenicity [11]. Additionally, influenza A NA is further classified into two groups: group 1 (N1, N4, N5 and N8) and group 2 (N2, N3, N6, N7 and N9), based upon primary sequence [12]. Group 1 NAs contain a 150-cavity (formed by amino acids 147–151 of the 150-loop) in their active site, whereas group 2 NAs lack this cavity [12]. Coordination of the 150-loop with the 430-loop appears to be critical for the formation of the 150-cavity [13], [14]. Soaking experiments of typical group 1 NAs with inhibitors often result in the closure of the 150-cavity and indicates some flexibility of the 150-loop [12], [15]. Molecular dynamics simulations also indicate some differences in the flexibility of the 150-loop between group 1 and group 2 NAs [14], [16]. Structural studies reveal that Asp151 and Arg152 of the 150-loop form key interactions with the 4-group and N-acetyl group of common NA ligands, respectively. These two residues move away from the substrate in the open conformation of the 150-loop and closer upon ligand binding [17]. Therefore the 150-loop plays an essential role in substrate and inhibitor binding [15]. Furthermore, the 150-cavity is currently being successfully explored as a target for novel NA inhibitors [12], [18], [19], [20].

**Figure 1:**
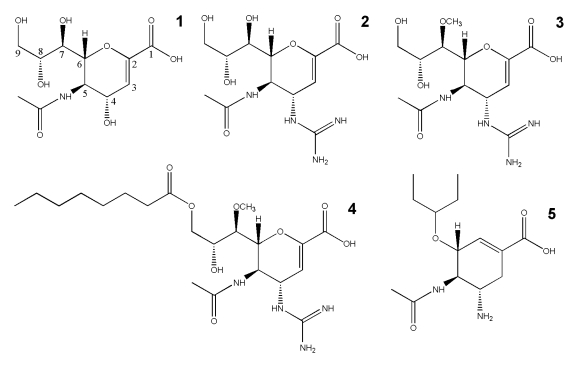
The chemical structures of influenza NA inhibitors used in this study. 1, Neu5Ac2en (NA transition state analogue); 2, zanamivir; 3, laninamivir; 4, laninamivir octanoate (CS-8958); and 5, oseltamivir.

The design of NA inhibitors is a classic example of structure-based drug discovery, pioneered by Mark von Itzstein and colleagues with the advent of the N2, N9 and influenza B NA structures [5], [6], [8], [21], [22], [23], [24]. Currently there are four NA-targeting inhibitors that have been approved for use: zanamivir, oseltamivir, peramivir and laninamivir (laninamivir has recently been approved in Japan). Additionally, there are many more NA inhibitors under clinical trials or under vigorous development due to the public threat of seasonal and pandemic flu and the rise of drug-resistant viruses [18], [19], [25], [26], [27], [28]. However, previous results have indicated that inhibitors which are highly similar to the natural NA ligand, sialic acid, are less susceptible to the problem of drug-resistance [29], [30], [31]. This suggests that drugs like zanamivir, that are similar to sialic acid and its transition state analogue 2-deoxy-2,3-dehydro-N-acetylneuraminic acid (Neu5Ac2en or DANA), have an advantage over oseltamivir, which is less similar (Figure 1). However, zanamivir must be administered twice daily over 5 consecutive days to attain its maximum effect. Therefore, the development of novel inhibitors that possess long term efficacy and that are also effective against oseltamivir-resistant influenza viruses is in great demand.

Laninamivir (R-125489) is a very promising, novel influenza NA inhibitor with high potency and the ability to efficiently inhibit common oseltamivir-resistant viruses, including those with the ubiquitous His274Tyr substitution [32], [33], [34]. Recently, laninamivir and its prodrug, laninamivir octanoate (CS-8958) have been approved for use in Japan as Inavir (Daiichi Sanko Press Release, 10 Sept. 2010). Clinical studies have confirmed that the prodrug, laninamivir octanoate, is effective in both children and adults, however laninamivir octanoate has not yet been demonstrated to be more effective than oseltamivir against oseltamivir-resistant His274Tyr H1N1 infection in adult patients [35], [36], [37]. Like zanamivir, the core structure of laninamivir is Neu5Ac2en, the NA transition state analogue (Figure 1). Both laninamivir and zanamivir contain a 4-guanidino group that is not present in Neu5Ac2en and laninamivir also contains an additional 7-methoxy group (Figure 1). Laninamivir octanoate is the octanoyl prodrug of laninamivir (Figure 1). In a similar manner that oseltamivir is processed to oseltamivir carboxylate in the liver, it has been demonstrated that laninamivir octanoate is processed to laninamivir in the lung [33]. The laninamivir 7-methoxy and its prodrug octanoyl ester increase the ability to be retained in the lungs and to function effectively in a single inhaled dose [32], [33], [34], [37], [38]. Moreover, the high similarity of laninamivir to the NA transition state analogue, Neu5Ac2en, allows for an effective response against oseltamivir-resistant NA [32], [33], [34], [35]. For these reasons, laninamivir and laninamivir octanoate offer advantages over both oseltamivir and zanamivir.

In order to comprehensively assess the effectiveness of the novel NA inhibitors, laninamivir and laninamivir octanoate, in comparison to oseltamivir and zanamivir, and to reveal the structural basis of their inhibition, we utilized: 1) pandemic A/RI/5+/1957 H2N2 N2 (p57N2) as a typical group 2 NA, 2) p09N1 as an atypical group 1 NA, and 3) avian H12N5 NA (N5) as a typical group 1 NA. Soluble, active p57N2, p09N1 and N5 were expressed in a baculovirus expression system and purified based upon previously reported methods [13], [39], [40]. NA inhibition assays were carried out and complex crystal structures were solved for laninamivir, laninamivir octanoate, zanamivir and oseltamivir in order to elucidate the structural basis of their inhibition. Our results indicate that laninamivir is potent against all 3 NAs with a similar binding mode to zanamivir. Laninamivir and zanamivir were more effective against group 1 N5, with a 150-cavity, than atypical group 1 p09N1 and group 2 p57N2, with no 150-cavity. This indicates that the ability of the bulky 4-guanidino group of zanamivir and laninamivir to become buried deep beneath the 150-loop is an important factor for their group-specific binding and inhibition. Furthermore, we confirm the binding of the prodrug, laninamivir octanoate, to p57N2, with a similar binding mode to laninamivir. Surprisingly, the p09N1-laninamivir octanoate complex shows a completely different binding mode: p09N1 adopts a Glu276-Arg224 salt bridge in its laninamivir octanoate complex, forming a hydrophobic pocket that is also necessary to accommodate oseltamivir. The observation of different Glu276 rotation in p09N1 and p57N2 offers insight into the group specific differences of oseltamivir binding and resistance.

## Results

### Comparison of the active sites of p57N2, p09N1 and N5

In our previous studies, we have successfully obtained both soluble p09N1 and N5 using a baculovirus expression system originally developed by Xu et al. [13], [15], [39]. To determine the functional and structural basis of NA inhibition and binding by laninamivir and its prodrug, we first expressed and purified a new group 2 member from the 1957 pandemic H2N2 virus, p57N2, using similar methods. In this way, three major types of known NAs are covered in this comprehensive analysis: typical group 2 p57N2, atypical group 1 p09N1 and typical group 1 N5.

**Figure 2:**
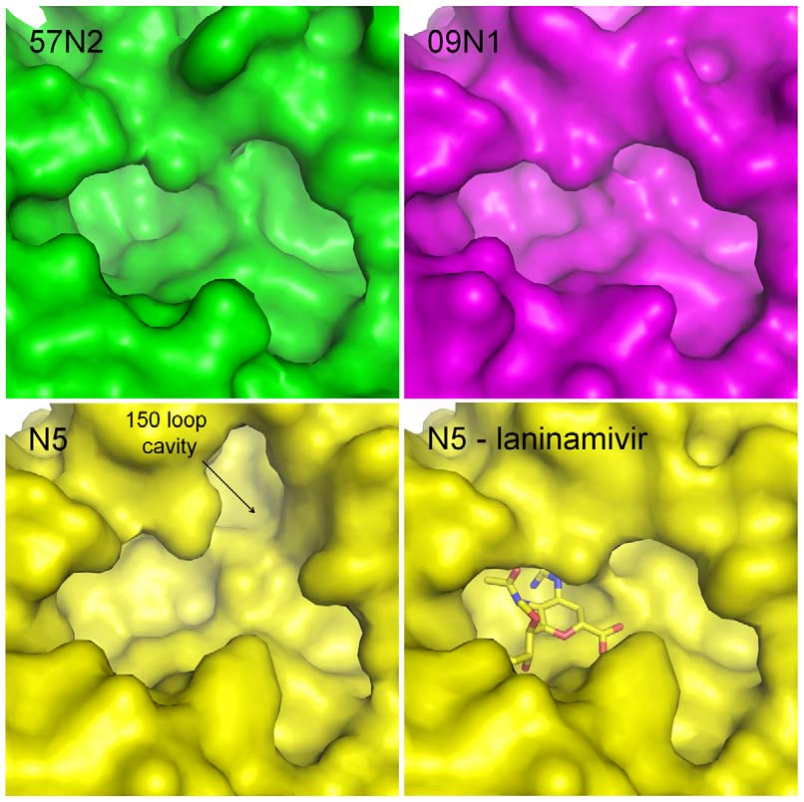
Comparison of the active sites of p57N2, p09N1 and N5. Group 2 p57N2 has a 150-cavity deficient active site with a salt bridge between Asp147 and His150 which stabilizes the closed conformation of its 150-loop (upper left - green). p09N1 is an atypical group 1 structure and also has a 150-cavity deficient active site similar to many group 2 structures (upper right - magenta). N5 is a typical group 1 NA and displays a 150-cavity in its uncomplexed structure (lower left - yellow) that closes upon inhibitor binding (lower right - yellow: N5-laninamivir complex).

**Figure 3:**
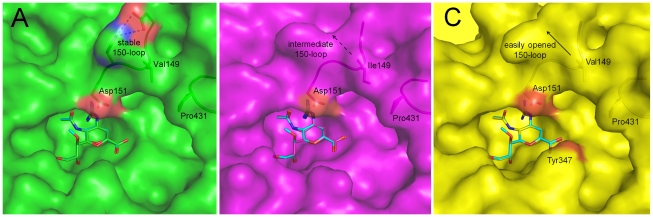
Binding of laninamivir and zanamivir to p57N2, p09N1 and N5. In each panel, zanamivir appears as the same color as the respective NA active site and laninamivir appears as turquoise. Acidic and basic side chains of key residues are colored red and blue, respectively. The 4-guanidino group of laninamivir and zanamivir is buried deep beneath the 150-loop where it engages many key interactions with NA residues (Table 2). Although the binding modes of laninamivir and zanamivir are highly similar, the accessibility of the 4-guanidino to its binding site is lowest in p57N2, with a 147–150 salt bridge in its closed 150-loop (A - green) and highest in group 1 N5, which contains a 150-cavity in its uncomplexed structure (C - yellow). Inhibition by zanamivir and laninamivir are highest for N5, and lowest for p57N2. p09N1, with its unique 150-loop characteristics (B - magenta), has intermediate laninamivir inhibition.

p57N2 was crystallized and its structure was solved by molecular replacement using A/TOKYO/3/1967 (H2N2) N2 (PDB code: 1IVG) as a search model [41]. As expected, the active site of p57N2 is highly similar to other group 2 NAs in that it has no 150-cavity (Figure 2 - upper left). Like the available group 2 A/TOKYO/3/1967 (H2N2) N2 and A/Memphis/31/98 (H3N2) N2 structures [42], [43], p57N2 also contains a 150-cavity deficient active site with a salt bridge between Asp147 and His150, confirming the presence of a stable, closed 150-loop (Figure 2 - upper left; Figure 3A).

Although the atypical group 1 p09N1 also has a 150-cavity deficient active site (Figure 2 - upper right), the 150-loop is quite different from that of p57N2. The p09N1 150-loop sequence (residues 147–150) is **G**T**IK**D, however p57N2 contains **D**T**VH**D with 3 polymorphic amino acids. The p09N1 therefore contains no Asp147-His150 salt bridge, but instead contains Ile149, which is commonly found in group 2 NAs, and Ile149 is able to rest closer to the hydrophobic Pro431 than Val149 is [13]. N5 on the other hand contains Val149 with no 147–150 salt bridge and displays a 150-cavity like all other structure-known NAs with Val149 and no 147–150 salt bridge (Figure 2 - lower left) [15]. Therefore, NAs with the three major styles of the 150-loop are covered in our comparative analysis.

### Differential inhibition of N5, p09N1 and p57N2 by oseltamivir, zanamivir, laninamivir and laninamivir octanoate

All NA proteins produced in the baculovirus expression system displayed stable sialidase activity. IC_50_ values and 95% confidence intervals (CIs) are given in Table 1. Oseltamivir inhibited the activity of N5, p09N1 and p57N2 with IC_50_ values of 0.83 nM, 0.54 nM and 0.79 nM, respectively. Laninamivir inhibition was best for group 1 N5, followed by atypical group 1 p09N1 and worst for group 2 p57N2. Zanamivir was also more effective against N5 than p09N1 and p57N2, however the difference of zanamivir inhibition between p09N1 and p57N2 is not statistically significant (Table 1). Zanamivir inhibited N5, p09N1 and p57N2 with IC_50_ values of 0.59 nM, 1.11 nM and 1.36 nM, respectively. Laninamivir was in a similar range with zanamivir for N5, p09N1 and p57N2 with IC_50_ values of 0.90 nM, 1.83 nM and 3.12 nM, respectively. However inhibition of laninamivir was 1.53, 1.65 and 2.29 fold lower than zanamivir for N5, p09N1 and p57N2, respectively. Inhibition of N5, p09N1 and p57N2 by laninamivir octanoate was not as efficient, with IC_50_ values of 389 nM, 947 nM, and 129 nM, respectively. Hence inhibition of p57N2 by laninamivir octanoate was much better than for p09N1.

**Table 1:** IC_50_ values and 95% CIs for the inhibition of p57N2, 09N1 and N5.

	p57N2		p09N1		N5	
	**IC_50_**	**95% CI**	**IC_50_**	**95% CI**	**IC_50_**	**95% CI**
**oseltamivir**	0.79	0.64 - 0.98	0.54	0.38 - 0.78	0.83	0.62 - 1.10
**zanamivir**	1.36	1.11 - 1.66	1.11	0.74 - 1.66	0.59	0.40 - 0.86
**laninamivir**	3.12	2.39 - 4.09	1.83	1.46 - 2.29	0.90	0.64 - 1.28
**CS-8958**	129	98 - 171	947	751 - 1192	389	329 - 459

Laninamivir octanoate is listed as CS-8958 to save space.

### Group specific NA binding of laninamivir and zanamivir

To determine the structural basis of the inhibition of laninamivir relative to zanamivir, we solved the very first complex structures of laninamivir with p57N2, p09N1 and N5 at resolutions of 1.8 Å, 1.8 Å and 1.6 Å, respectively; and the complex structures of zanamivir with p57N2, p09N1 and N5 at 1.9 Å, 1.9 Å and 1.6 Å, respectively [15]. Like zanamivir, the binding mode of laninamivir to all 3 NAs is highly similar to that of the NA transition state analogue, Neu5Ac2en. Some minor differences in the NA-inhibitor interactions between laninamivir and zanamivir are observed within each of the 3 NAs due to the additional hydrophobic 7-methoxy group of laninamivir; however all of the laninamivir complex structures highly resemble zanamivir binding (Table 2, Figure 3).

**Table 2:** Comparison of key NA-ligand interactions.

		p57N2 Distance			p09N1 Distance			N5 Distance	
Protein Group	Ligand Group	zanamivir	laninamivir	CS-8958	zanamivir	laninamivir	CS-8958	zanamivir	laninamivir
R118	carboxy	2.83/3.14	2.87/3.28	2.96/3.42	2.76/3.46	2.75/3.60	2.83/3.64	2.81/3.51	2.77/3.62
E119	4-X	3.33/3.64	3.26/3.76	3.20/4.03	3.22/3.77	3.24/3.93	3.28/3.88	3.30/3.94	3.27/3.90
D151*	4-X	2.94	2.90	3.00	2.96	2.89	2.92	2.91	2.79
D151**	4-guan	3.15	2.95	2.93	3.01	2.88	2.96	2.93	2.91
R152	5-Ac	2.87	2.84	2.86	2.86	2.82	2.82	2.83	2.88
W178**	4-guan	2.71/3.07	2.68/3.00	2.86/2.99	2.73/3.11	2.69/2.95	2.79/3.16	2.82/3.18	2.76/3.17
R224δN	9-O	3.37	3.41	3.09	3.33	3.45	-	3.43	3.24
R224δN	9-ester***	-	-	3.52	-	-	-	-	-
E227	4-guan	3.03	3.09	3.48	3.12	3.06	3.04	3.02	3.02
S247	9-ester-O	-	-	-	-	-	3.50	-	-
E276	8-OH	2.80	2.76	2.71	2.72	2.63	-	2.65	2.68
E276	9-O	2.65	2.61	2.80	2.59	2.61	-	2.52	2.59
R292	carboxy	3.36/3.43	3.27/3.29	3.26/3.49	3.19/3.24	3.09/3.16	3.14/3.12	3.14/3.19	3.09/3.11
R292	8-OH	3.52/3.52	3.50/3.62	3.56/3.73	3.43/3.73	3.58/3.82	3.46/3.48	3.64/3.89	3.56/3.71
N294	9-ester-O	-	-	-	-	-	2.64,3.93	-	-
R371	carboxy	2.71/2.79	2.76/2.77	2.43/2.81	2.70/2.94	2.67/2.83	2.80/2.95	2.89/2.93	2.74/2.80
Y406	ring-O	3.00	3.14	2.96	3.14	3.15	3.36	3.25	3.03

All residues are N2 numbered and distances are given in Å. The 4-guanidino group of zanamivir, laninamivir and CS-8958 is abbreviated as ‘4-guan’. Bond distances are based on the distances between oxygen and nitrogen atoms and do not include hydrogen atoms, which cannot be directly observed using X-ray diffraction. Distances are given for molecule A in the asymmetric unit of each structure and are highly consistent between molecules. The distance of the unique hydrogen bond between the laninamivir octanoate 9-ester-O and p09N1 Asn294 is given for both molecules A and B as it varies significantly. Laninamivir octanoate is listed as CS-8958 to save space.

*The Asp151 side chain carboxy hydrogen bonds with the 4-N of all the ligands used in this study.

**The Asp151 and Trp178 backbone carbonyl groups both hydrogen bond with the 4-guanidino group of zanamivir, laninamivir and laninamivir octanoate.

***The laninamivir octanoate 9-ester-carbonyl forms a hydrogen bond with N2 Arg224.

Due to the similar binding modes of zanamivir and laninamivr, we first carried out a detailed analysis of interactions with the 150-loop in each inhibitor complex. In all of the zanamivir and laninamivir structures, the 4-guanidino group is buried deep beneath the 150-loop where it forms many key hydrogen bonds with Glu119, the Trp178 peptide carbonyl, Glu227, and the Asp151 side chain and peptide carbonyl (Figure 3, Table 2). This 4-guanidino group is the most buried part of the inhibitor in the structure (Figure 3), which is emphasized by the absence of any water molecules beneath the 150-loop and surrounding the 4-guanidino group. Although the 4-guanidino plays an essential role for the high affinity of laninamivir and zanamivir to NA, accessibility of the 4-guanidino to its binding site deep below the 150-loop is a crucial factor for the laninamivir and zanamivir binding process. The typical group 1 N5 contains a 150-cavity in its uncomplexed structure and inhibition of N5 by laninamivir and zanamivir was better than inhibition of p09N1 and p57N2, which contain no 150-cavity in their uncomplexed structures (Figure 3, Table 1). Therefore, our data indicate that the group specific accessibility of the laninamivir and zanamivir 4-guanidino to the NA active site is a key factor in determining their effectiveness.

Slight differences in the interactions between the binding of laninamivir and zanamivir were observed due to the additional laninamivir 7-methoxy group (Table 2). Although this laninamivir 7-methoxy group is oriented away from its own ring oxygen and is pointed toward the hydrophobic Ile222 side chain, its distance is relative far at over 5 Å. Interactions between Arg371 and the inhibitor carboxylate were always highly consistent; however the carboxylate-Arg118 interactions are closer in zanamivir than laninamivir in every NA complex (Table 2). On the other hand, the carboxylate-Arg292 interactions are further in zanamivir than laninamivir in every NA complex (Table 2). Unlike p09N1 and p57N2, N5 contains Tyr347, which forms an additional hydrogen bond with the carboxylate of each inhibitor (Figure 3C).

### Differential binding of the octanoate prodrug to p57N2 and p09N1 via enhanced p09N1 Glu276 rotation

**Figure 4:**
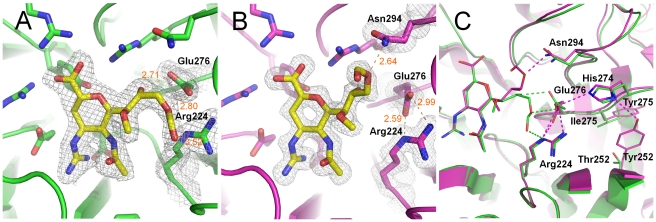
Binding of the laninamivir octanoate prodrug (CS-8958) to p57N2 and p09N1. A) Binding of laninamivir octanoate to p57N2, which is similar to laninamivir/zanamivir NA binding, except with a novel hydrogen bond between the 9-ester carbonyl and Arg224. B) Unique binding mode of laninamivir octanoate to p09N1. Glu276 is rotated to form a salt bridge with Arg224 and there is a novel hydrogen bond between the laninamivir octanoate 9-ester-O and Asn294. C) Comparison of laninamivir octanoate binding to p57N2 and p09N1. Key residues are labeled. Tyr252 is group 1 specific and takes part in a hydrogen bond network with His274 and Glu276. The less bulky Thr252 (compared to group 1 Tyr252) is group 2 specific and allows for greater movement of residue 274 away from Glu276. The His274Tyr substitution results in group 1 specific oseltamivir-resistance also via Glu276 interactions.

**Figure 5:**
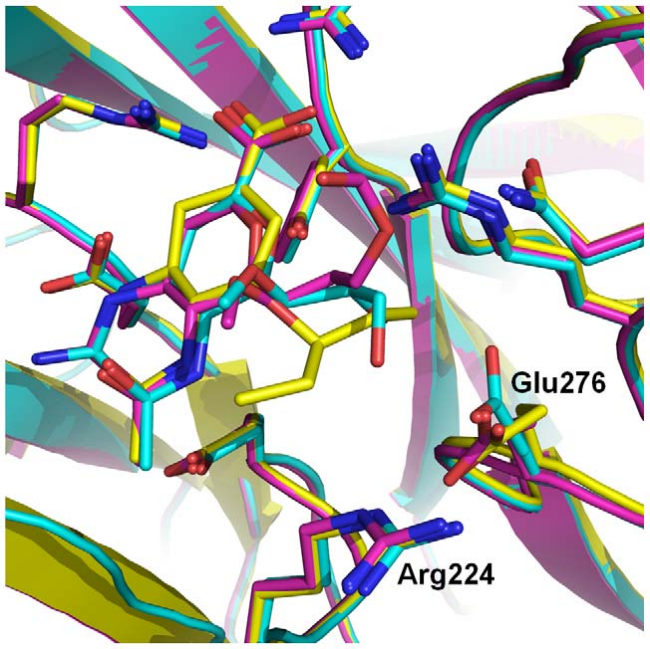
Comparison of oseltamivir (yellow), laninamivir octanoate (magenta) and laninamivir (turquoise) binding to p09N1. Laninamivir binds to p09N1 with a similar active site conformation to the uncomplexed structure. Both laninamivir octanoate and oseltamivir binding to p09N1 induces rotation of Glu276 toward Arg224 where they form a salt bridge. This Glu276 rotation creates a hydrophobic pocket that accommodates the hydrophobic pentyl ether side chain of oseltamivir, however results in a weaker overall binding mode of laninamivir octanoate. The terminal carbon of the oseltamivir side chain is 3.73 Å from the hydrophobic Glu276 Cβ.

Laninamivir octanoate complex structures with p09N1 and p57N2 (Figure 4) were solved at 1.6 Å and 2.2 Å, respectively, demonstrating that the laninamivir octanoate prodrug can also directly inhibit NA without further processing. In p57N2, laninamivir octanoate binds in a similar manner to laninamivir with an additional, novel hydrogen bond between the 9-ester carbonyl and Arg224 (Figure 4A). p09N1, on the other hand, has a totally different binding mode where the prodrug's ester is oriented toward Asn294 rather than Arg224 (Figure 4B). p09N1 Glu276 is also in a different orientation in the laninamivir octanoate complex structure than in the zanamivir or laninamivir complex structures and forms a salt bridge with Arg224 in the same manner as oseltamivir binding (Figure 4 and 5). The rotation of p09N1 Glu276 places it out of range for hydrogen bonding with the 8-OH and 9-ester-O of laninamivir octanoate. Instead, the p09N1-laninamivir octanoate 9-ester-O forms a unique hydrogen bond with Asn294 (Figure 4B). Additionally, 09N1 Ser247 forms another hydrogen bond with the laninamivir octanoate 9-ester-O at 3.4–3.5 Å. Still, the Glu276 rotation results in less hydrogen bonding in the p09N1-laninamivir octanoate complex compared to p57N2 (Figure 4C, Table 2).

In both structures, there is no observed electron density corresponding to the octanoyl carbon chain indicating that this part of the molecule is highly flexible and does not engage many stable hydrophobic interactions with p09N1 or p57N2. Still, electron density surrounding the entire ester can be observed in both complex structures. Furthermore, in p09N1, the position 7-methoxy of laninamivir octanoate is also oriented slightly away from its N-acetyl group relative to laninamivir and there is additional electron density pointing toward the ring, indicating lower stability of the p09N1-laninamivir octanoate complex (Figure 4). In all of our NA complex structures, bond distances in each molecule of the asymmetric units are very similar, however in the p09N1-laninamivir octanoate structure some greater differences are observed between molecule A and B in the asymmetric unit, which further reflects the lower stability of the prodrug's octanoyl ester in p09N1. In p09N1 molecule A, the distance between the 9-ester-O and Asn294 is 2.64 Å, however in molecule B the distance is much further at 3.93 Å (Table 2).

### An oseltamivir-like binding mode of laninamivir octanoate with p09N1

To our surprise, the binding mode of the p09N1-laninamivir octanoate complex structure is similar to all known NA-oseltamivir complex structures with respect to the Glu276-Arg224 interactions. Therefore, we also solved the p09N1 oseltamivir complex structure at a resolution of 1.7 Å. As observed in the other available oseltamivir-NA complex structures, in the p09N1-laninamivir octanoate complex structure, Glu276 indeed forms a salt bridge with Arg224, creating a hydrophobic pocket which accommodates the hydrophobic oseltamivir pentyl ether group (Figure 5) [7], [29], [44], [45]. This hydrophobic side chain of oseltamivir is favorably parallel to the Cβ and Cγ of Glu276 on one end and at the other end is pointed toward the hydrophobic Ile222 side chain, which contributes significantly to the high level of oseltamivir inhibition.

## Discussion

Recent studies have demonstrated that the novel influenza A virus NA inhibitors, laninamivir and laninamivir octanoate, are highly effective and have some advantages over zanamivir and oseltamivir [33], [34], [35], [37], [38]. In this study we verify that laninamivir, which highly resembles the NA transition state analogue, Neu5Ac2en, is indeed effective at inhibiting highly purified p57N2, p09N1 and N5, representing the three major types of all structure-known NAs with distinct 150-loop properties.

Zanamivir and laninamivir are clearly more similar to sialic acid and Neu5Ac2en, than oseltamivir, which renders zanamivir and laninamivir less susceptible to drug-resistance and effective against many oseltamivir-resistant viruses [29], [30], [31]. The high degree of similarity between the binding modes of zanamivir and laninamivir in all of the NA complex structures indicates that zanamivir and laninamivir should be effective against the same drug-resistant mutations. However, laninamivir contains an additional 7-methoxy group which is oriented toward Ile222. Although the distance between the laninamivir 7-methoxy and Ile222 is relative far (over 5 Å), laninamivir may be susceptible to Ile222Arg, a rare drug-resistant substitution [46]. Moreover, the additional 7-methoxy group of laninamivir may disrupt hydrogen bonding of the 7-O with water and likely contributes to a slightly lower inhibition of laninamivir compared to zanamivir that was observed for all 3 NAs here (Table 1).

Although zanamivir and laninamivir are highly similar to Neu5Ac2en, they both contain an artificial bulky 4-guanidino group. Upon binding, this 4-guanidino group becomes buried deep beneath Asp151 of the closed 150-loop and forms many hydrogen bonds which contribute to the high affinity of zanamivir and laninamivir to NA. However, the bulky 4-guanidino must be able to clear the 150-loop in order to bind NA and therefore a closed 150-loop may hinder the entry of zanamivir and laninamivir into the NA active site. In the open state of the 150-loop, when the 150-cavity is formed, Asp151 shifts over 1.5 Å (the Asp151 Cγ is shifted over 2 Å in N5) away from the ligand binding site, which should facilitate entry of inhibitors like zanamivir and laninamivir [12], [15], [17]. A similar model has recently been proposed by Wang et al., however this was based on a computer simulation using only the group 1 H5N1 NA structure [47].

The group specific 150-loop accessibility, based upon our structures of p57N2, p09N1, and N5, is consistent with the inhibition efficiency of laninamivir and zanamivir. Group 2 p57N2 contains an Asp147-His150 salt bridge, limiting the flexibility of its closed 150-loop and inhibition of p57N2 by laninamivir was the lowest (Figure 3A). p09N1 is an atypical group 1 with no 150-cavity, but no Asp147-His150 salt bridge, and inhibition of p09N1 by laninamivir was better than p57N2 (Figure 3B). The typical group 1 N5 contains a 150-cavity in its uncomplexed structure and inhibition of N5 by both laninamivir and zanamivir was the highest (Figure 3C). Therefore, we provide structural and functional evidence that the open 150-loop of a typical group 1 NA may facilitate the entry of the 4-guanidino group of zanamivir and laninamivir into the NA active site, relative to the closed 150-loop of group 2 NAs. The additional hydrogen bond between Tyr347 and the inhibitor carboxylate is also a key factor in explaining the higher N5 inhibition relative to p09N1 and p57N2. However, like the closed 150-loop, this residue also makes the active site cavity smaller and in this way may also limit access of inhibitors to the N5 active site. Furthermore, this residue is found only in group 1 NAs which contain an open 150-loop cavity [12]. Thus, Tyr347 may compensate for the open 150-loop in regards to substrate binding.

The complex structure of p57N2 with the laninamivir octanoate prodrug has a similar binding mode to laninamivir and zanamivir, however laninamivir octanoate in complex with p09N1 is completely different. This is the first time, as far as we know, that the same inhibitor has been observed to bind in two completely different conformations to influenza NAs. Additionally, p57N2 Arg224 forms a unique hydrogen bond with the laninamivir octanoate 9-ester carbonyl, and p09N1 Asn294 and Ser247 form unique hydrogen bonds with the laninamivir octanoate 9-ester-O. However, the novel conformation of the laninamivir octanoate-p09N1 complex disrupts any hydrogen bonding with Glu276. The overall lack of hydrogen bonds and instability in the p09N1-laninamivir octanoate structure relative to p57N2 provides the structural basis for higher laninamivir octanoate inhibition of p57N2 observed in our study and a previous report demonstrating better laninamivir octanoate inhibition of H2N2 and H3N2 viruses over H1N1 viruses [34]. The absence of any electron density surrounding the octanoyl carbon chain of laninamivir octanoate indicates that it is unable to take part in any favorable interactions with p57N2 and p09N1. The disordered octanoyl carbon chain likely destabilizes the interactions between the NA active site and the laninamivir octanoate 8-OH and 9-ester, which is indicated by the lower electron density surrounding the 9-ester. Therefore, the lower inhibition efficiency of laninamivir octanoate relative to laninamivir is not surprising.

Binding of oseltamivir to p09N1 was indeed highly similar to the binding observed in previous reports and is also similar to the binding mode of laninamivir octanoate to p09N1. Oseltamivir contains a 4-amino group, instead of the 4-guanidino group found in zanamivir and laninamivir, and is actually more similar to the natural ligand in this regard. Therefore, the orientation of the 150-loop during oseltamivir binding is not a major factor. Instead, the binding preference of oseltamivir for p09N1 over p57N2 and N5 may be instead explained by the ability of Glu276 to adopt the conformation that is critical to accommodate the osetalmivir pentyl ether side chain, which replaces the glycerol moiety of zanamivir, laninamivir and sialic acid. The observation that this Glu276 conformation occurs in the p09N1-laninamivir octanoate complex, but not the p57N2-laninamivir octanoate complex may indicate that this conformation is more stable in p09N1 after ligand binding which may explain why inhibition of p09N1 by oseltamivir was the best relative to N5 and p57N2.

In addition, this observation of different Glu276 dynamics in group 1 p09N1 compared to group 2 p57N2 offers some new insights into the group specificity of the oseltamivir-resistant His274Tyr substitution. The His274Tyr mutation is easily selected for N1 viruses, however cannot be selected for N2 virus types as N2 His274Tyr binding to oseltamivir is not impaired [48]. In group 2 NAs, Tyr274 is able to move away from Glu276 due to a small neighboring Thr252 residue, and oseltamivir can still bind for His274Tyr [30]. The native His274 is also further away from Glu276 in our p57N2-laninamivir octanoate structure and does not hydrogen bond with it. In group 1 NA, Tyr274 is not able to move away from Glu276 because of the bulky neighboring Tyr252 side chain, which prevents it from accommodating oseltamivir [30]. In a similar manner, the group 1 Tyr252 side chain promotes the native His274 to occupy a position where it can participate in a hydrogen bond network with Glu276 and Arg224 as observed in our 09N1-laninamivir octanoate structure (Figure 4C).

Recently, a clinical study has shown that laninamivir octanoate is not significantly better than oseltamivir against oseltamivir-resistant His274Tyr H1N1 infection in adult patients [36]. Since the laninamivir octanoate prodrug binds to p09N1 in a similar manner to oseltamivir, this may offer some explanation as to why laninamivir octanoate has a similar effect as oseltamivir against His274Tyr H1N1. However, this may indicate that the laninamivir octanoate is not processed, or processed slowly, to laninamivir in the adult patients from this study, since laninamivir has been clearly demonstrated to be effective against the oseltamivir-resistant His274Tyr influenza A viruses [32], [33], [34]. Further investigation into the efficacy of laninamivir octanoate in adults in clearly needed.

The results from this comprehensive analysis of group 2 p57N2, atypical group 1 p09N1 and typical group 1 N5 support the hypothesis that influenza NA inhibitors which more closely resemble the NA transition state analogue, Neu5Ac2en, are more likely to remain effective against NAs from both groups and with various drug-resistant amino acid substitutions. Most importantly, we provide mechanisms to explain the group 1 preference of laninamivir and zanamivir and the differential binding of the octanoate prodrug to group 1 p09N1 and group 2 p57N2 derived from pandemic influenza viruses.

## Materials and Methods

### Reagents

Methylumbelliferyl-N-acetylneuraminic acid (MUNANA) was purchased from J&K Scientific Ltd. Sialic acid (Neu5Ac) was purchased from Sigma (Cat. No. 855650) and used without further purification. Laninamivir, laninamivir octanoate, zanamivir and oseltamivir were readily synthesized according to the relevant literatures [49], [50], [51], [52], [53]. All products were characterized by their NMR or MS spectra.

### Expression and purification of recombinant NAs

NA was prepared in a baculovirus expression system according to methods based on an original method reported by Xu et al. [39]. Both N5 and p09N1 were prepared as previously described in our laboratory [13], [15]. For p57N2, the cDNA encoding amino acid residues 83–469 were recombined into the baculovirus transfer vector pFastBac1 (Invitrogen), with a GP67 signal peptide, a 6X his-tag, a tetramerizing sequence and a thrombin cleavage site at the N-terminus. Recombinant baculovirus was prepared based on the manufacturer's protocol (Invitrogen). Sf9 suspension cultures were grown in Sf-900 II SFM serum-free media (GIBCO) at 28°C and 120 RPM and transfected with high-titer recombinant baculovirus. After growth of the transfected Sf9 suspension cultures for 3 days, centrifuged media were applied to a HisTrap FF 5 mL column (GE Health) which was washed with 20–50 mM imidazole, and then NA was eluted using 200–300 mM imidazole. After dialysis, thrombin digestion (Sigma, 3 U/mg NA; overnight at 4°C) and gel filtration chromatography using a Superdex-200 10/300 GL column (GE Healthcare), NA fractions were analyzed by SDS-PAGE. High-purity NA fractions were pooled and concentrated using a membrane concentrator with a molecular weight cutoff of 10 kD (Millipore). A buffer of 20 mM Tris-HCl, 50 mM NaCl, pH 8.0 was used for gel filtration and protein concentration.

### Competition experiments

A neuraminidase inhibition assay using MUNANA was performed as described by Potier et al. with modifications [54]. Briefly, 10 uL of purified, recombinant NA (10 nM) was mixed with 10 uL of inhibitor and incubated for 30 min at room temperature. NA and inhibitors were carefully diluted in fresh PBS buffer. At least 5 concentrations of each inhibitor at an appropriate range were used for each repeat. Following incubation, 30 uL of 166 uM MUNANA in 33 mM MES and 4 mM CaCl_2_ (pH 6.0) was added to the solution to start the reaction using a 12-tip pipette (Eppendorf). A positive and a negative control were included in each 12-well lane. After starting the reaction for each lane on the plate, the reaction mixture was immediately loaded on a SpectraMax M5 (Molecular Devices) where fluorescence was quantified over the course of 30 min at an excitation wavelength of 355 nm and an emission wavelength of 460 nm. Single time points were chosen where the positive control produced a fluorescence signal of approximately 1,000. All assays were done in triplicates and IC_50_ values for each inhibitor were calculated with sigmoidal fitting of the log[inhibitor] vs. inhibition percentage using GraphPad Prism.

### Crystallization and drug soaking

NA crystals were grown using the hanging-drop vapor diffusion method. Initial screening was performed using a commercial kit (Hampton Research). Diffraction quality crystals of p57N2 were obtained by mixing 1 uL of the concentrated protein at 10 mg/mL in 20 mM Tris, pH 8.0, and 50 mM NaCl with 0.1M BIS-TRIS propane (pH 9.0), 10% v/v Jeffamine ED-2001 (pH 7.0). N5 crystals were obtained using 0.1 M HEPES (pH 7.5), 12% w/v polyethylene glycol 3,350 at 18°C [15]. Quality p09N1 crystals were obtained as described previously using 0.16 M calcium acetate hydrate, 0.08 M sodium cacodylate trihydrate, pH 6.5, 14.4% polyethylene glycol 8000, 20% glycerol at 18°C [13]. NA protein crystals were first incubated in mother liquor containing 20 mM of inhibitor, and then flash-cooled at 100 K. Diffraction data for the p57N2 and N5 were collected at KEK beamline Ne3A, while p09N1 data were collected at SSRF beamline BL17U.

### Data collection, processing and structure solution

Diffraction data were processed and scaled using HKL2000 [55]. Data collection and processing statistics are summarized in Table 3. The structure of p57N2 was solved by molecular replacement method using Phaser [56] from the CCP4 program suite [57] with the structure of A/TOKYO/3/1967 H2N2 N2 (PDB code: 1IVG) as the search model [41]. Initial restrained rigid-body refinement and manual model building were performed using REFMAC5 [58] and COOT [59], respectively. Further rounds of refinement were performed using the phenix.refine program implemented in the PHENIX package with coordinate refinement, isotropic ADP refinement and bulk solvent modeling [60]. The stereochemical quality of the final model was assessed with the program PROCHECK [61]. The final models have 84% of the residues in the most favored region of the Ramachandran plot [62] and no residue in disallowed regions. Structures of p09N1 and N5 were solved as described previously [13], [15].

**Table 3:** Crystallographic X-ray diffraction and refinement statistics.

	N2-zanamivir	N2-lanamivir	N2-CS-8958	09N1-zanamivir	09N1-laninamivir	09N1-CS-8958	09N1-oseltamivir	N5-lanamivir
**Data collection**								
Space group	P21	P21	P21	C2221	C2221	C2221	C2221	P4
Cell dimensions								
* a*, *b*, *c* (Å)	90.13	89.93	90.45	118.45	118.26	118.67	118.24	112.56
	140.00	140.31	140.18 90.30	136.96	137.09	137.10	137.16	112.56
	90.17	89.89		118.47	118.48	118.57	118.65	66.81
α, β, γ (°)	90, 101.3, 90	90,101.5,90	90, 101.2, 90	90, 90, 90	90, 90, 90	90, 90, 90	90, 90, 90	90, 90, 90
Resolution (Å)	50-1.90 (1.97-1.90)	50-1.80 (1.86-1.80)	50-2.20 (2.28-2.20)	50-1.90 (1.97-1.90)	50-1.80 (1.86-1.80)	50-1.60 (1.66-1.60)	50-1.70(1.76-1.70)	50-1.60(1.66-1.60)
*R*_merge_	0.120 (0.396)	0.126 (0.551)	0.162 (0.491)	0.155 (0.443)	0.147 (0.531)	0.103 (0.317)	0.135 (0.519)	0.082 (0.554)
*I*/σ*I*	11.0 (2.1)	12.7 (3.0)	9.6 (3.3)	12.8 (4.7)	16.5 (3.6)	20.6 (6.2)	17.1 (4.0)	22.1 (2.8)
Completeness (%)	98.7 (90.0)	99.9 (99.9)	99.9 (99.9)	99.4 (99.6)	99.9 (99.7)	99.6 (99.0)	100.0 (100.0)	100.0 (100.0)
Redundancy	4.1 (3.6)	4.1 (4.1)	3.9 (4.0)	6.5 (5.5)	10.6 (9.6)	7.1 (6.8)	8.1 (7.0)	6.1 (5.8)
**Refinement**								
Resolution (Å)	41.27-1.89	42.99-1.80	43.17-2.20	36.13-1.90	34.60-1.80	25.90-1.60	30.00-1.69	37.80-1.60
No. reflections	160592	190839	103790	71738	86190	122842	103720	105988
*R*_work_/*R*_free_	0.1531/0.1838	0.1516/0.1698	0.1548/0.1910	0.1673/0.1933	0.1938/0.2135	0.1400/0.1767	0.1387/0.1833	0.1268/0.1606
No. atoms								
Protein	12380	12380	12397	6175	6098	6114	6098	6194
Ligand/ion	96	100	108	51	53	57	45	50
Water	2154	1934	1394	765	1301	1278	802	1162
*B*-factors								
Protein	15.2	13.4	14.3	11.9	9.9	8.2	9.9	14.1
Ligand/ion	12.9	9.5	12.7	13.2	9.6	9.9	10.5	10.5
Water	31.8	31.2	28.4	29.7	26.7	31.4	29.8	33.7
R.m.s. deviations								
Bond lengths (Å)	0.005	0.006	0.006	0.003	0.004	0.004	0.003	0.004
Bond angles (°)	1.030	1.127	0.984	0.840	0.935	1.069	0.842	1.008
Ramachandran plot								
Most favored (%)	85.7	86.1	84.9	85.2	84.7	85.5	85.3	87.7
Additionally favored (%)	13.6	13.1	14.2	14.5	15.0	13.9	14.4	11.9
Generally allowed (%)	0.8	0.8	0.8	0.3	0.3	0.6	0.3	0.4
Disallowed (%)	0	0	0	0	0	0	0	0

Laninamivir octanoate is listed as CS-8958 to save space.

### PDB accession codes

All crystal structures have been deposited into the Protein Data Bank (PDB, www.pdb.org) with the following PDB codes: N5-laninamivir - 3TI8, p09N1-zanamivir - 3TI5, p09N1-laninamivir - 3TI3, p09N1-laninamivir octanoate - 3TI4, p09N1-oseltamivir - 3TI6, p57N2-zanamivir - 3TIC, p57N2-laninamivir - 3TIA, and p57N2-laninamivir octanoate - 3TIB.
